# The Role of Preoperative Radiological, Cytological, and Systemic Inflammatory Markers in Predicting Incidental Papillary Microcarcinoma in Patients with Multinodular Goiter: A Retrospective Single-Center Study

**DOI:** 10.3390/jcm15114343

**Published:** 2026-06-04

**Authors:** Yavuz Selim Kahraman, Fatma Gün, İbrahim Kurtoğlu, Yasin Alper Yıldız, Ezgi Kartal, Adem Şentürk

**Affiliations:** 1 Department of General Surgery, Division of Surgical Oncology, Kastamonu Training and Research Hospital, Kastamonu 37150, Türkiye; 2 Department of General Surgery, Kastamonu University, Kastamonu 37150, Türkiye; fcetin@kastamonu.edu.tr (F.G.); ikurtoglu@kastamonu.edu.tr (İ.K.); yalperyildiz@gmail.com (Y.A.Y.); 3 Department of Radiology, Yeditepe University, Istanbul 34755, Türkiye; ezgikartal@yeditepe.edu.tr; 4 Department of Surgical Oncology, Sakarya Training and Research Hospital, Adapazarı 54290, Türkiye; dr.adem.senturk@gmail.com

**Keywords:** multinodular goiter, papillary thyroid microcarcinoma, neutrophil-to-lymphocyte ratio, systemic immune-inflammation index, thyroid imaging reporting and data system (TI-RADS), Bethesda system for reporting thyroid cytopathology

## Abstract

**Objective**: Preoperative prediction of incidental papillary thyroid microcarcinoma (PTMC) in patients undergoing surgery for multinodular goiter (MNG) is a clinically significant challenge. This study aimed to evaluate the association of preoperative radiological (TI-RADS), cytological (Bethesda), and systemic inflammatory markers (neutrophil/lymphocyte ratio [NLR] and systemic immune-inflammation index [SII]) with incidental PTMC. **Methods**: This retrospective study included patients who underwent total thyroidectomy due to multinodular goiter. Preoperative ultrasonography findings (TI-RADS), fine needle aspiration biopsy results (Bethesda), and inflammatory markers (NLR and SII) were analyzed. Patients were grouped according to the presence of PTMC based on postoperative pathology results. Independent associations with PTMC were evaluated using multivariable binary logistic regression analysis with TI-RADS and Bethesda classifications entered as categorical variables. **Results**: A total of 283 patients who underwent total thyroidectomy for multinodular goiter were included in the study. The mean age was 52.75 ± 11.39 years, and 78.1% of the patients were female. Postoperative histopathological examination revealed incidental papillary thyroid microcarcinoma (PTMC) in 67 patients (23.6%). PTMC was detected in 67 patients (23.6%). In multivariable binary logistic regression analysis, high-risk TI-RADS categories (TR4–5 vs. TR1–3) and Bethesda III–IV categories (vs. Bethesda I–II) were independently associated with PTMC, whereas NLR and SII were not independently significant. **Conclusions**: High-risk TI-RADS and Bethesda categories were independently associated with incidental PTMC in patients with multinodular goiter, whereas inflammatory markers including NLR and SII were not independent predictors. Radiological and cytological evaluation appear to remain the primary components of preoperative assessment in incidental PTMC.

## 1. Introduction

Papillary thyroid carcinoma (PTC) is the most common form of endocrine malignancy. Its global incidence has significantly increased in recent years, mainly due to the widespread adoption of high-resolution imaging techniques and the routine use of fine-needle aspiration biopsy (FNAB) to evaluate thyroid nodules [1]. Many newly diagnosed PTCs are papillary thyroid microcarcinomas (PTMCs), which are defined as malignant epithelial tumors with a maximum diameter of 1 cm or less. They are often found incidentally during histological examination following thyroidectomy [2]. PTMCs are frequently detected as incidental findings during postoperative histopathological analysis of thyroidectomy specimens obtained for presumed benign thyroid conditions, especially multinodular goiter (MNG) [3,4]. The prevalence of incidental papillary thyroid microcarcinoma in benign thyroidectomy ranges from approximately 10% to 20%, and in larger sample sizes, this rate is around 15–17% [5,6].

MNG remains a common surgical indication in endocrinology practice; however, identifying concomitant malignancy before surgery remains difficult and limited. Conventional imaging with thyroid ultrasonography helps stratify malignancy risk, but when multiple nodules are present, accurately targeting the most suspicious lesion can be difficult, thereby reducing the diagnostic performance of standardized systems [7]. The American College of Radiology Thyroid Imaging Reporting and Data System (TI-RADS) provides a standardized risk-assessment algorithm that uses ultrasound features—such as nodule composition, echogenicity, shape, margins, and echogenic foci—to assess the likelihood of thyroid cancer. Although TI-RADS has enhanced diagnostic reproducibility and serves as a helpful framework in routine clinical practice, its sensitivity and specificity in patients with multinodular thyroid disease and in detecting small, incidental carcinomas remain limited. This limitation is significant, given the American Thyroid Association’s recommendation for risk-based management strategies tailored to individual sonographic profiles, which emphasizes careful evaluation in complex nodular settings [8].

In recent years, hematological inflammatory markers such as the neutrophil-to-lymphocyte ratio (NLR), platelet-to-lymphocyte ratio (PLR), and systemic immune-inflammation index (SII) have become affordable, non-invasive diagnostic adjuncts for the preoperative evaluation of thyroid malignancies. A 2023 meta-analysis demonstrated that these inflammatory indices have significant diagnostic value in papillary thyroid carcinomas and are associated with aggressive tumor characteristics [9]. In particular, the systemic immune-inflammation index (SII) is a reliable predictor of central lymph node metastasis in PTMC cases lacking clinical evidence of nodal involvement, with a high area under the curve (AUC) of 0.803 [10]. Furthermore, NLR is strongly correlated with aggressive pathological features, including capsular invasion, increased tumor diameter, and microvascular invasion, in PTMC [11]. A separate 2022 study demonstrated that combining NLR and PLR with TI-RADS scoring significantly enhanced diagnostic accuracy for malignant thyroid nodules in the preoperative setting [12].

Despite advances in imaging and cytopathology, comprehensive studies evaluating the combined diagnostic utility of preoperative ultrasonography, cytological findings, and systemic inflammatory markers in patients undergoing thyroidectomy for MNG with incidental PTMC detection remain scarce in the literature. Therefore, this study aims to assess the preoperative predictability of incidentally detected PTMC and evaluate it by integrating these diagnostic methods.

## 2. Materials and Methods

### 2.1. Study Design and Setting

This retrospective, single-center observational study enrolled adult patients who underwent total thyroidectomy for multinodular goiter (MNG) at Kastamonu University Faculty of Medicine between January 2022 and July 2025. Ethical approval for this study was obtained from the Kastamonu University Faculty of Medicine Non-Interventional Clinical Research Ethics Committee (date: 14 July 2025, approval number: 2025-19) and the study was conducted in accordance with the principles of the Declaration of Helsinki (1964). Due to the retrospective design of the study and the nature of data collection, informed consent was not deemed necessary.

### 2.2. Study Population

A total of 283 patients who underwent total thyroidectomy were included in the study. A post hoc power analysis was performed to evaluate the adequacy of the sample size. The analysis indicated that a minimum sample size of approximately 240 patients would provide 80% statistical power at a significance level of 0.05, and the included sample size of 283 patients was therefore considered adequate. Patients with incidental papillary thyroid microcarcinoma (PTMC) identified during postoperative histopathological evaluation constituted the case group. A control group with comparable baseline demographic and clinical characteristics, including age, sex, and nodule characteristics, was included, comprising patients undergoing thyroidectomy for benign MNG during the same period. This study employed a retrospective sequential cohort design. Patients with papillary thyroid microcarcinoma (PTMC) incidentally detected histopathologically after surgery formed the study group, while patients without PTMC formed the control group.

### 2.3. Inclusion Criteria and Exclusion Criteria

Inclusion criteria included patients aged ≥18 years who had preoperative thyroid ultrasonography (USG), fine-needle aspiration biopsy (FNAB), and complete blood count (CBC) data. All participants had histopathologically confirmed MNG (with or without PTMC) and complete accessible medical records, including imaging, cytological, and laboratory results. While both devices are safe for surgical procedures, both the harmonic scalpel (HS) and the LigaSure vessel (LS) have been used during thyroidectomy depending on intraoperative conditions and the surgeon’s preference. Incidental PTMC was defined as patients with papillary thyroid microcarcinoma who were detected only during postoperative histopathological examination, and surgery was partly driven by cytology. Exclusion criteria included a preoperative diagnosis of thyroid cancer (Bethesda V–VI), a history of hematologic malignancies, systemic inflammatory conditions, immunosuppressive therapy, extrathyroidal malignancies, active infections, and incomplete or missing preoperative records. Patients undergoing subtotal thyroidectomy or lobectomy were also excluded. Bethesda V–VI were excluded because they strongly suggest malignancy. Bethesda categories III–IV were included in the study because they belong to the uncertain cytology group. Bethesda III–IV was included in the primary analysis because it represents indeterminate malignant cytology. In the group without PTMC, Bethesda categories I–II were more predominantly identified. A significant number of PTMC cases were also detected in Bethesda I–II patients; this supports the idea that these lesions are incidentally observed in a clinically low-risk cytological subgroup.

### 2.4. Data Collection

Ultrasound features (such as number, size, echogenicity, margins, calcifications, and composition of nodules) were reviewed and categorized according to the ACR TI-RADS classification. Thyroid ultrasonography findings were evaluated according to the 2017 American College of Radiology (ACR) TI-RADS classification system, using a composite score-based TR1–TR5 categorization approach. TI-RADS classifications were retrospectively derived from original radiology reports. Ultrasonographic examinations and TI-RADS evaluations were performed by multiple experienced radiologists. The radiologists were unaware of the postoperative histopathological outcomes at the time of the original examination.

FNAB outcomes were reported based on the Bethesda System for Reporting Thyroid Cytopathology. Hematological indices were calculated as follows:

- Neutrophil-to-Lymphocyte Ratio (NLR): Neutrophil count/Lymphocyte count
- Platelet-to-Lymphocyte Ratio (PLR): Platelet count/Lymphocyte count
- Systemic Immune-Inflammation Index (SII): (Platelet count × Neutrophil count)/Lymphocyte count

All hematological parameters were obtained from preoperative CBC results processed in the institutional laboratory.

### 2.5. Statistical Analysis

Statistical analyses were performed using IBM SPSS Statistics (version 26.0). The distribution was evaluated using the Kolmogorov–Smirnov test. Continuous variables were presented as mean ± standard deviation, and categorical variables as number and percentage. Student’s *t*-test, chi-square test, or Fisher’s test were used to compare groups based on the normality of the distributions. Multivariable binary logistic regression was performed to evaluate factors independently associated with incidental PTMC. TI-RADS and Bethesda classifications were performed as categorical variables. So, TI-RADS categories were grouped as TR1–3 versus TR4–5. Bethesda categories were grouped as Bethesda I–II versus Bethesda III–IV. Results were reported as odds ratios with 95% confidence intervals. Statistical significance was accepted as *p* < 0.05.

## 3. Results

### 3.1. Demographic Characteristics Between Groups

**Figure 1:**
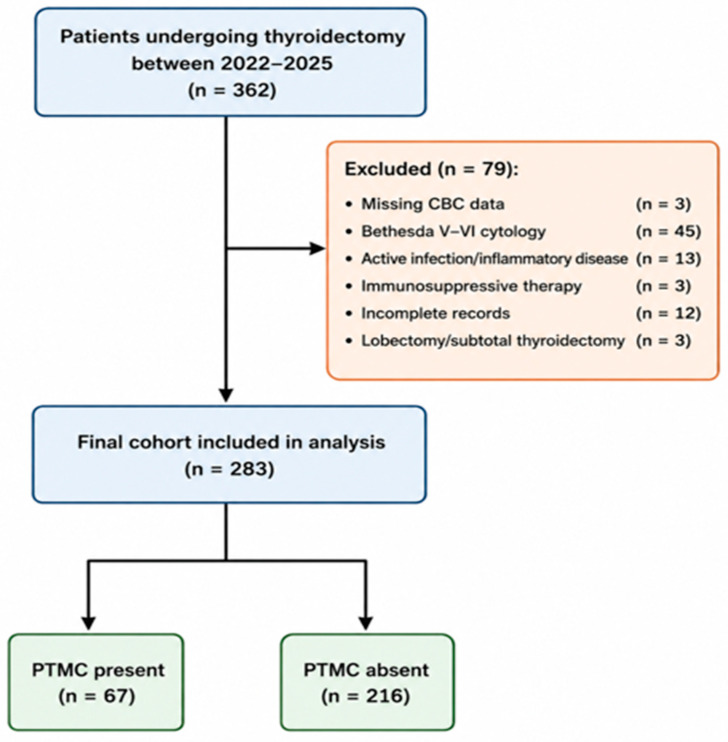
Flow diagram of patient selection.

A total of 283 patients who underwent total thyroidectomy due to multinodular goiter were included in the study. The patient selection process is summarized in Figure 1. The mean age was 52.75 ± 11.39 years, and out of the 283 patients, 62 (21.9%) were male and 221 (78.1%) were female. Postoperative histopathological examination revealed papillary thyroid microcarcinoma (PTMC) in 67 patients (23.6%). There was no statistically significant difference in gender distribution between the groups with and without PTMC (*p* = 0.570) (Table 1).

### 3.2. Bethesda Distribution Between Groups

In the PTMC and non-PTMC groups, Bethesda I was detected in 10 (14.9%) and 42 (19.4%) patients, respectively; Bethesda II was detected in 17 (25.4%) and 94 (43.5%); Bethesda III was detected in 18 (26.9%) and 41 (19.0%); and Bethesda IV was detected in 22 (32.8%) and 39 (18.1%), respectively. Bethesda III–IV categories were more common in the PTMC group, while Bethesda I–II categories were more common in the non-PTMC group. A significant number of PTMC cases were also detected in patients with Bethesda I–II cytology; this indicates the incidental nature of tumors in a clinically low-risk cytological subgroup (Table 2).

### 3.3. Comparison of Variables Between Patients with or Without PTMC

When clinical, radiological, and hematological parameters were compared, no significant differences were observed between the groups in age or TSH levels (*p* = 0.405 and *p* = 0.817, respectively). Preoperative ultrasonography demonstrated significantly smaller nodule size in the PTMC group compared with the non-PTMC group (25.94 ± 16.08 mm vs. 32.35 ± 16.77 mm; *p* = 0.008). Patients with PTMC were significantly more likely to have high-risk TI-RADS categories (TR4–5) than patients without PTMC (55.2% vs. 29.2%, *p* < 0.001). Similarly, Bethesda III–IV categories were more common in the PTMC group than in the non-PTMC group (59.7% vs. 37.0%, *p* = 0.002). Among the hematological parameters, NLR and SII values were significantly higher in patients with PTMC (*p* = 0.020 and *p* = 0.018, respectively), whereas neutrophil count, lymphocyte count, platelet count, and PLR did not differ significantly between the groups (all *p* > 0.05) (Table 3).

### 3.4. Comparison of Clinical, Ultrasonographic, and Laboratory Parameters According to Histopathological Features

In subgroup analyses, the presence of microcalcifications was significantly associated with high-risk TI-RADS and Bethesda categories. Tumor diameter (*p* = 0.001), TSH level (*p* = 0.044), preoperative nodule size (*p* < 0.001), and nodule localization (*p* = 0.042) were found to be significantly associated with the tumor. No significant associations were observed between categorized TI-RADS or Bethesda groups and multifocality, capsular invasion, Hashimoto’s thyroiditis, or concurrent thyroiditis (all *p* > 0.05). Similarly, hematological parameters, including neutrophil count, lymphocyte count, platelet count, NLR, PLR, and SII, were not significantly associated with any histopathological features (all *p* > 0.05) (Table 4).

### 3.5. Multivariable Binary Logistic Regression Analysis for PTMC

Multivariable binary logistic regression analysis showed that high-risk TI-RADS categories (TR4–5 vs. TR1–3) and Bethesda III–IV categories (vs. Bethesda I–II) were independently associated with PTMC. In contrast, NLR and SII were not independently significant predictors. In addition, no significant association was found between age and male gender and incidental PTMC (*p* > 0.05). (Table 5).

## 4. Discussion

Preoperative identification of incidental papillary thyroid microcarcinoma (PTMC), which is incidentally detected in the preoperative period in patients undergoing surgery for multinodular goiter, is a clinically challenging issue. In this study, while high-risk TI-RADS and Bethesda categories were found to be independently associated with PTMC, systemic inflammatory markers such as NLR and SII did not show an independent association with PTMC. Compared with subtotal thyroidectomy, total thyroidectomy may be associated with a slightly higher risk of early postoperative complications. In the present study, no patient required sternotomy, and all retrosternal goiters were successfully managed via a cervical approach. However, sternotomy is rarely necessary and reserved for selected complex cases.

In this study, the incidence of random PTMC was found to be slightly above the upper limit of 23.6%, similar to large sample studies. This can be explained by the exclusion of Bethesda V–VI cases and extensive histopathological sampling [5,6,13]. Most papillary microcarcinomas are clinically silent and are detected at high rates in autopsy studies [14]. However, some cases may progress, and factors such as age may influence disease course [15]. Accordingly, active surveillance has been shown to be a safe management option in selected low-risk PTMC cases [16]. In the context of multinodular goiter, clinical evaluation typically focuses on the larger, dominant nodules, which may lead to malignant foci in smaller nodules being overlooked, thereby contributing to the limited preoperative detection of incidental PTMC [17]. Furthermore, certain patients may present with more aggressive features, such as lymph node metastasis, which can significantly impact clinical decision-making and management strategies in multinodular goiter [18].

TI-RADS and Bethesda are the most widely used approaches for evaluating malignancy risk [19,20]. In our study, high-risk TI-RADS categories (TR4–5) and Bethesda III–IV categories were more frequently in patients with incidental PTMC. The fact that they were found to be independently associated with PTMC in multivariate regression analysis demonstrates the importance of radiological and cytological examinations in preoperative evaluation.

Although the role of inflammation in cancer development is known, its clinical impact can vary depending on the size of the tumor and the stage of the disease [21,22,23]. Inflammatory diseases such as Hashimoto’s thyroiditis have been shown to contribute to the development of thyroid cancer by altering the immune environment around the tumor [21]. In our study, NLR and SII values were found to be significantly higher in the PTMC group in univariate analyses; however, they were not significant as independent determinants in the multivariate regression model. This finding suggests that the additional contribution of systemic inflammatory markers to preoperative evaluation may be limited, especially in incidental PTMC cases with low tumor burden and slower clinical course. Similarly, the literature has shown that inflammatory markers are more strongly associated with aggressive clinicopathological features, lymph node metastasis, and advanced thyroid cancer than with incidental microcarcinoma [10,22,23,24,25]. This result highlights the key role of imaging and cytology-based risk stratification in preoperative evaluation of patients with multinodular goiter.

### Limitations

There are several limitations associated with the present study: the design was retrospective, and the study was single-center. The retrospective and single-center design limits the generalizability of the results. Additionally, multicollinearity among certain radiological, cytological, and inflammatory variables might have impacted the stability of the multivariable model. Internal validation (bootstrap or k-fold cross-validation) was not performed due to the retrospective single-center design. In addition, the lack of follow-up data, single-center design with a single laboratory, and exclusion of Bethesda V–VI cases may limit the generalizability and external validity of the findings. Because TI-RADS classifications were retrospectively derived from original radiology reports, inter-observer agreement was not involved. Additionally, although TI-RADS and Bethesda classifications are categorical in multivariate analyses, analyzing them as categorical variables by grouping them according to high or low risk may have reduced the impact of risk classification.

## 5. Conclusions

In conclusion, high-risk TI-RADS and Bethesda categories were independently associated with incidental PTMC in patients undergoing thyroidectomy. The central role of radiological and cytological assessment in preoperative risk stratification is important. In contrast, systemic inflammatory markers, including NLR and SII, failed to maintain their independent significance in multivariate analysis. This suggests that they may offer limited additional benefit in this clinical setting. Further prospective multicenter studies with standardized risk stratification protocols are needed to confirm these findings and clarify the potential complementary role of inflammatory biomarkers in incidental PTMC.

## Figures and Tables

**Table 1:** Demographic characteristics between groups.

Demographic Characteristics	PTMC Present	PTMC Absent	Total
Age (mean ± SD)	51.76 ± 11.53	53.06 ± 11.02	52.75 ± 11.39
Male, *n* (%)	13 (19.4)	49 (22.7)	62 (21.9%)
Female, *n* (%)	54 (80.6)	167 (77.3)	221 (78.1%)
Total, *n* (%)	67 (100%)	216 (100%)	283 (100%)

**Abbreviations:** PTMC, papillary thyroid microcarcinoma; %, percent; SD, standard deviation.

**Table 2:** Bethesda distribution between groups.

Bethesda	PTMC Present *n* (%)	PTMC Absent *n* (%)	Total (*n*)
I	10 (14.9)	42 (19.4)	52
II	17 (25.4)	94 (43.5)	111
III	18 (26.9)	41 (19.0)	59
IV	22 (32.8)	39 (18.1)	61
Total	67 (100)	216 (100)	283

**Abbreviations:** PTMC, papillary thyroid microcarcinoma; %, percent.

**Table 3:** Comparison of Variables between Patients with or without PTMC.

Variables	PTMC Present (67) Mean ± SD	PTMC Absent (216) Mean ± SD	*p* Value
Age (years)	51.76 ± 11.53	53.06 ± 11.02	0.405
TSH (uIU/mL)	2.15 ± 3.94	2.02 ± 3.95	0.817
Preop USG Nodule Size (mm)	25.94 ± 16.08	32.35 ± 16.77	0.008
TI-RADS TR1–3 *n* (%)	30 (44.8)	153 (70.8)	<0.001
TI-RADS TR4–5 *n* (%)	37 (55.2)	63 (29.2)	
FNAB Bethesda I–II *n* (%)	27 (40.3)	136 (63.0)	0.002
FNAB Bethesda III–IV *n* (%)	40 (59.7)	80 (37.0)	
Neutrophils (10^3^/µL)	4.92 ± 1.78	4.55 ± 1.64	0.109
Lymphocytes (10^3^/µL)	2.22 ± 0.79	2.30 ± 0.67	0.437
Platelets (10^3^/µL)	289.96 ± 87.45	273.38 ± 74.76	0.129
NLR	2.46 ± 1.31	2.10 ± 1.02	0.020
PLR	138.91 ± 42.48	119.65 ± 27.56	0.165
SII	695.09 ± 378.66	578.13 ± 344.70	0.018

**Abbreviations:** PTMC, papillary thyroid microcarcinoma; SD, standard deviation; TSH, thyroid-stimulating hormone; USG, ultrasonography; TI-RADS, Thyroid Imaging Reporting and Data System; FNAB, fine-needle aspiration biopsy; NLR, neutrophil-to-lymphocyte ratio; PLR, platelet-to-lymphocyte ratio; SII, systemic immune-inflammation index.

**Table 4:** Comparison of clinical, ultrasonographic, and laboratory parameters according to histopathological features.

Variable	Micro Calcification *n* = 54 * vs. *n* = 229 **	Multi Focality *n* = 32 * vs. *n* = 38 **	Capsular Invasion *n* = 9 * vs. *n* = 61 **	Hashimoto’s Thyroiditis *n* = 4 * vs. *n* = 67 **	Concurrent Thyroiditis *n* = 26 * vs. *n* = 47 **
Age	0.067	0.730	0.307	0.332	0.297
TI-RADS TR4–5 vs. TR1–3	<0.001	0.587	0.660	0.711	0.987
Bethesda III–IV vs. I–II	0.008	0.179	0.373	0.843	0.927
Neutrophil (10^3^/µL)	0.396	0.616	0.693	0.928	0.947
Lymphocyte (10^3^/µL)	0.969	0.376	0.755	0.565	0.195
Platelet (10^3^/µL)	0.791	0.436	0.985	0.332	0.169
NLR	0.775	0.376	0.672	0.575	0.389
PLR	0.065	0.655	0.127	—	0.083
SII	0.848	0.875	0.927	0.832	0.068
Tumor Diameter (mm)	0.001	0.258	0.247	0.855	0.874
TSH (uIU/mL)	0.044	0.346	0.526	0.430	0.496
Preoperative USG Nodule Size (mm)	<0.001	0.494	0.672	0.909	0.195
Nodule Localization	0.042	0.085	0.559	0.707	0.555

**Abbreviations:** *, present; ** absent; NLR, neutrophil-to-lymphocyte ratio; PLR, platelet-to-lymphocyte ratio; SII, systemic immune-inflammation index; USG, ultrasonography; TSH, thyroid-stimulating hormone. **Note:** Student’s *t*-test or Mann–Whitney U test for continuous variables and chi-square or Fisher’s exact test for categorical variables. “—” indicates data not available.

**Table 5:** Multivariable Binary Logistic Regression Analysis Associated with PTMC.

Variables	OR	95% CI	*p*-Value
Age	0.996	0.970–1.023	0.789
Male sex	0.925	0.443–1.932	0.836
TI-RADS TR4–5 vs. TR1–3	2.733	1.484–5.033	0.001
Bethesda III–IV vs. I–II	1.943	1.070–3.527	0.029
NLR	1.405	0.873–2.262	0.161
SII (per 100-unit increase)	0.982	0.847–1.139	0.813

**Abbreviations:** PTMC, papillary thyroid microcarcinoma; OR, odds ratio; CI, confidence interval; NLR, neutrophil-to-lymphocyte ratio; SII, systemic immune-inflammation index; TI-RADS, Thyroid Imaging Reporting and Data System.

## Data Availability

The datasets generated and/or analyzed during the current study are not publicly available due to privacy but are available from the corresponding author upon reasonable request.

## Abbreviations

The following abbreviations are used in this manuscript:

PTMC	Papillary Thyroid Microcarcinoma
SD	Standard Deviation
TSH	Thyroid-Stimulating Hormone
USG	Ultrasonography
TI-RADS	Thyroid Imaging Reporting And Data System
FNAB	Fine-Needle Aspiration Biopsy
NLR	Neutrophil-To-Lymphocyte Ratio
PLR	Platelet-To-Lymphocyte Ratio
SII	Systemic Immune-Inflammation Index
MPV	Mean Platelet Volume
RDW	Red Cell Distribution Width
AUC	Area Under The Curve
CI	Confidence Interval
OR	Odds Ratio
